# Epigallocatechin-3-gallate ameliorates hepatic damages by relieve FGF21 resistance and promotion of FGF21–AMPK pathway in mice fed a high fat diet

**DOI:** 10.1186/s13098-022-00823-y

**Published:** 2022-04-13

**Authors:** Yuanyuan Zhang, Ruili Yin, Jianan Lang, Ying Fu, Longyan Yang, Dong Zhao

**Affiliations:** Beijing Key Laboratory of Diabetes Research and Care, Center for Endocrine Metabolism and Immune Diseases, Beijing Luhe Hospital, Capital Medical University, Beijing, 101149 China

**Keywords:** NAFLD, EGCG, FGF21–AMPK

## Abstract

**Background:**

Non-alcoholic fatty liver disease (NAFLD) is considered to be one of the most common chronic liver diseases across worldwide. Epigallocatechin-3-gallate (EGCG) derived from extract of green tea and is well known for beneficial effects on anti-oxidative, anti-inflammatory, and anti-tumor activity. The present study aimed to implore its underlying mechanism for protective effect of NAFLD.

**Methods:**

Mice were fed either high fat diet (HFD) or chow diet with or without EGCG treatment in HFD group, for up to 16 weeks. Histopathology, expression of lipid and glucose metabolism and lipogenesis-related gene expression were assessed. Primary mouse hepatocytes were treated with free fatty acids combined with different doses of EGCG for 48 h, expression of lipid and lipogenesis-related gene expression were assessed.

**Results:**

The results showed that EGCG attenuated HFD- and FFA-induced lipid accumulation in vivo and in vitro. EGCG can decrease the oxidative stress and promote Nrf2 level. Meanwhile EGCG alleviated FGF21 resistance and elevated FGFR/AMPK expression, which suggested an unrecognized mechanism of EGCG in ameliorating NAFLD.

**Conclusions:**

EGCG attenuated hepatocytes damage and dysfunction in NAFLD by alleviating FGF21 resistance and improve FGFR/AMPK pathway, mitigating oxidative stress. Our studies verified that EGCG may become a promising drug to treat or relieve NAFLD.

## Introduction

Non-alcoholic fatty liver disease (NAFLD) is currently considered as the most common chronic liver diseases in coming years with a prevalence of approximately 25–30% worldwide [1]. NAFLD encompasses different pathologies ranging from uncomplicated hepatic fat accumulation (simple steatosis) to a state of lobular inflammation and hepatocyte ballooning, known as non-alcoholic steatohepatitis (NASH), liver fibrosis, cirrhosis and hepatocelluar carcinoma [2]. Patients with NASH experience accelerated hepatic fibrosis and up to 20% will develop irreversible fibrosis (cirrhosis), ultimately requiring liver transplantation [3, 4]. The problem originated from NAFLD caused huge burden to human health and social economy. Finding ways to reverse or alleviate NAFLD are critical.

Green tea is a popular beverage consumed by a lot of people around the world and it has been widely researched. Epigallocatechin gallate (EGCG), as the main active ingredient of green tea, has various biological effects such as scavenging free radicals, anti-lipid peroxidation, anti-inflammatory, anti-viral, and enhancing the immune function of the body. It has obvious pharmacological effects in preventing obesity, diabetes, atherosclerosis and other diseases [5, 6]. A lot of research had shown EGCG could attenuate high-fat diet- (HFD-) induced NAFLD in rats and mice [7, 8]. Nevertheless, the related mechanisms of EGCG to ameliorate fat accumulation or NAFLD are not clearly understood yet.

Fibroblast growth factor-21 (FGF21), as a member of the fibroblast growth factor family, is involved in many metabolic processes including insulin sensitivity, glucose and lipid metabolism, and energy homeostasis. As FGF21 is a liver derived, metabolically active hormone, it is conceivable that FGF21 is implicated in the pathobiology of NAFLD. There is an abundance of preclinical evidence demonstrating aberrant FGF21 signaling is, at least partially, responsible for the pathogenesis and progression of NAFLD [9, 10]. In human, observational studies have demonstrated circulating FGF21 levels are elevated in subjects with NAFLD [11]; animal experiments have shown that FGF21 mRNA is increased in the livers of mice with NAFLD [12]. Recent studies demonstrated that activation of peroxisome proliferator-activated receptor α (PPARα) increases FGF21 production in WAT and livers [12, 13].

AMP-dependent protein kinase (AMPK) has been considered as the fuel gauge of the cells or the natural energy sensor that regulates lipid metabolism [14]. Recent studies have revealed FGF21 can stimulate AMPK signaling in several tissues [15, 16]. Studies shown FGF21 regulated mitochondrial activity and lipid metabolism through stimulation of LKB-AMPK pathway in adipocytes and in white adipose tissue [17, 18]. Chau et al. demonstrated that LKB1 might be is one of the phosphorylation targets of FGFR.

In the study, to delineate the potential therapeutic effects of EGCG in preventing NAFLD, we given c57BL/6 mice an HFD diet with or without EGCG supplementation treatment until 16 weeks. We explored the underlying mechanism and particularly focused on the PPARα–FGF21–AMPK signaling and oxidative stress in prevention of NAFLD for EGCG.

## Materials and methods

Animals. Experimental protocols were approved by the Animal Care and Use Committee of Beijing Luhe Hospital, Capital Medical University and conducted in accordance with the NIH Guide for the Care and Use of Laboratory Animals. All efforts were made to minimize suffering of experimental mice in this research. Animal studies are reported in compliance with the ARRIVE guidelines. Animals were randomly divided into three groups after a week of adaptive feeding. The HFD composed of 45% energy as fat, 20% protein, and 35% carbohydrates (Medicience Biotechnology Co., Ltd, Jiangsu, China) was used to induce non-alcoholic fatty liver. Mice were divided three groups: a normal diet group (CON) (n = 10), a high-fat diet (HFD) (n = 12) and a high-fat diet supplemented with 1% EGCG (HFD + EGCG) (n = 12). EGCG was administered at dose of 100 mg/kg for 16 weeks. All mice were maintained on a 12 h light/dark cycle and allowed access to food and water ad libitum. Food/water intakes and body weights of the mice were measured. After mice were anesthetized using ketamine and xylazine, livers were harvested. Blood was collected from the heart, immediately frozen with liquid nitrogen, and stored at −80 °C.

### Biochemical analysis

The concentrations of triglyceride (TG), total cholesterol (TC), alanine aminotransferase (ALT), and aspartate aminotransferase (AST) in the plasma were examined by an automated hematology analyzer (BC-6900, Mindray, Shenzhen, Guangdong, China). The contents of TG and TC in hepatocytes and liver tissues in liver tissues were detected by commercial enzyme-linked immunosorbent assay kits (Jiancheng Bioengineering Institute, Nanjing, Jiangsu, China).

### Oil red O staining

Primary mouse hepatocytes (C57BL/6J, 6–8 weeks old) were plated into six-well plates and exposed to FFA (PA OA = 1:2) with or without EGCG for 48 h (n = 4). After fixation with 4% paraformaldehyde for 10 min, fixed cells were stained in ORO solution for 15 min, and then their nucleus was stained by hematoxylin for 3 min. Oil red O staining was used to visualize lipid droplets in the liver.

### Level of FGF21 expression

Blood samples were collected from the mice, and the plasma was isolated. Circulating FGF21 was measured using an enzyme-linked immunosorbent assay (ELISA) kit purchased from Jonln Bioscience (Shanghai, China). The cell culture medium was collected, and the level of FGF21 protein expression was measured using an ELISA kit and conducted following the manufacturer’s protocol.

### Measurement of oxidative stress markers and antioxidant enzymes

The level of liver lipid peroxidation product MDA was measurement by using a commercial kit (Applygen Technologies, Beijing, China). The activities of antioxidant defense enzymes including SOD were measured by Elisa kit (Enzyme-linked Biotechnology, Shanghai, China).

### Hematoxylin and eosin (HE) staining

Liver tissues were fixed in 4% paraformaldehyde, embedded in paraffin wax, Serial sections were sliced at 5 μm and stained with hematoxylin–eosin (H&E) to observe the damage.

### Real-time quantitative PCR

Total RNA from liver tissues and primary hepatocytes was extracted with TRIzol reagent (Invitrogen, USA) and then reverse-transcribed using a cDNA Synthesis Kit (Bio-Rad, USA, Cat: 1708891). Primers were designed with Primer Premier 6.0 software and chemically synthesized by Sangon Biotech (Shanghai, China) Co., Ltd. The forward and reverse primer sequences for mouse genes are listed in Table 1. Real-time PCR was conducted with the SYBR Green PCR kit (Bio-Rad, USA, Cat: 1725151), and quantification was achieved by normalization using 18 s. The primer sequences are provided in Table 1.

**Table 1:** Sequences of primer pairs used for amplification of mRNA by real time PCR

Target	Forward primer	Reverse primer
FGFR2	GCCTCTCGAACAGTATTCTCCT	ACAGGGTTCATAAGGCATGGG
FGFR3	TGGATCAGTGAGAATGTGGAGG	CCTATGAAATTGGTGGCTCGAC
FGFR4	GCTCGGAGGTAGAGGTCTTGT	CCACGCTGACTGGTAGGAA
SREBP1	ACTTCTGGAGACATCGCAAAC	GGTAGACAACAGCCGCATC
ACC1	TGGTCGTGACTGCTCTGTGC	GTAGCCGAGGGTTCAGTTCC
SCD1	ATGTGCCAGAGGAGCTGAGT	TGATCCACTGTTGCTTCTGC
Actin	CGTAAAGACCTCTATGCCAACA	CGGACTCATCGTACTCCTGCT
FAS	GCTGCGGAAACTTCAGGAAAT	AGAGACGTGTCACTCCTGGACTT
AMPK	TACTCAACCGGCGAG CG	AGACGGCGGCTTTCCTTTT
PPARα	AGGAAGCCGTTCTGTGACAT	TTGAAGGAGCTTTGGGAAGA
LKB1	TTCAACCGAGTGGGTTATTTTGT	TACTGGGCATCTGGGGTGTAG
Actin	CGTAAAGACCTCTATGCCAACA	CGGACTCATCGTACTCCTGCT

### Western blot

Western blot assay was applied to determine the expression levels of proteins from cultured cells and frozen liver samples. The primary antibodies were as follows: FGF21 (Abcam, UK, Cat: ab171941), phospho-Thr172 AMPKα (CST, USA, Cat: 2535), AMPKα (CST, USA, Cat: 2532), ACC (Santa Cruz, USA, Cat: sc137104), FASn (Abcam, UK, Cat: ab128870), Nrf2 (Abcam, UK, Cat: ab137550), Actin (Zsbio, China, Cat: TA-09). Band intensities were analyzed by densitometry using ImageJ software.

### Primary mouse hepatocytes culture and treatment

Primary mouse hepatocytes were cultured as Yang et al. reported previously PMID: 28411267. Firstly, perfuse the mouse liver with 50 mL Kreb solution, followed by 30 mL Kreb solution with 0.2 mL liberase TM (Roche, Germany) to digest the liver. The cells were filtered with a disposable cell strainer (Corning, USA) using 1640 medium containing 10% FBS; then washed and centrifuged at 50 *g* at 4 °C for three times. The cells were cultured with 1640 containing 10% FBS for 6 h at 37 °C with 5% CO_2_ before treating.

### Immunofluorescence analysis

Primary mouse hepatocytes were seeded onto glass cover slips in six-well plates and treated with FFA and EGCG for 48 h, washed with cold PBS, and fixed in 4% paraformaldehyde in PBS at room temperature. The fixed cells were washed three times with PBS and kept in chilled methanol for 10 min at −20 °C.The cells were washed three times and incubated with the primary antibody and secondary antibody (AlexaFluor goat anti-mouse 488, 1:500).Then, the nucleus was counter-stained blue with DAPI (4',6-diamidino-2-phenylindole) and mounted with anti-fluorescence quenching sealant. The slips were imaged using an inverted fluorescence microscope.

### CCK8 assay

Primary mouse hepatocytes were plated at a density of 5 × 10^4^ cells/mL into 96-well plates (100 µL medium/well) with five replicates. The hepatocytes cells were then treated with different doses of EGCG and FFA. After seeding for 48 h, cell viability was measured using a CCK8 assay according to the manufacturer's instructions.

### Statistics

All data were presented as the mean ± SD. Differences between the two groups were determined by the two-tailed Student’s t-test and one-way analysis of variance using GraphPad Prism 6 software. P < 0.05 was considered to indicate a statistically significant difference.

## Results

### EGCG decreased hyperlipidemia and glucose in HFD mice

**Fig. 1:**
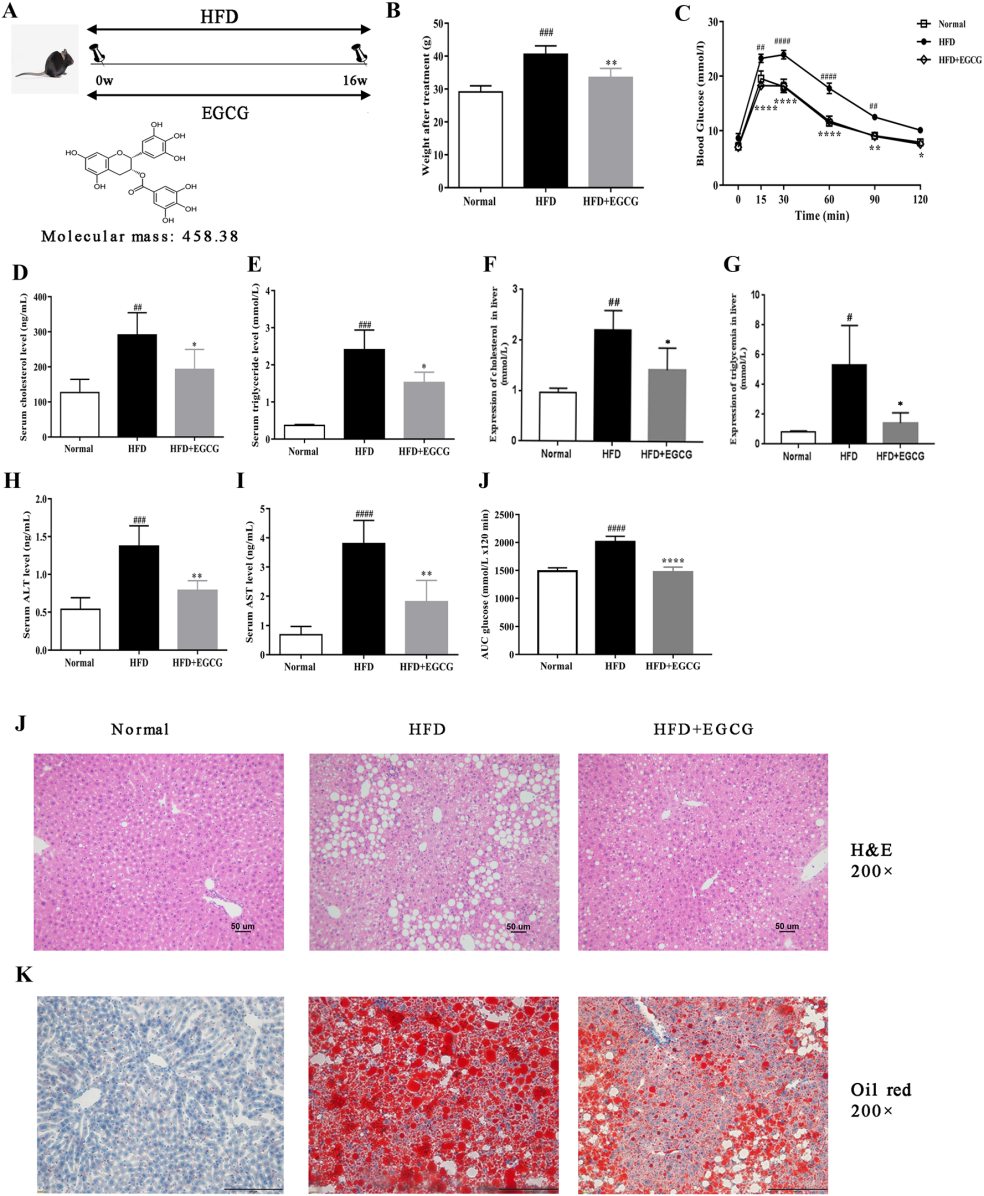
Effects of EGCG on body mass, biochemical levels in HFD-fed C57BL/6 mice. **A** Experimental flow chart; **B **effects of EGCG on body weight; **C** effects of EGCG on glucose levels; **D**, **E** effects of EGCG on serum TG and TC, **F**, **G **effects of EGCG on TG and TC in liver tissue, **H**, **I** serum ALT and AST levels, **J** IPGTT-AUC (area under the curve); **K** HE (× 200) of livers, **L** oil red O staining (× 200) of livers. ^#^p < 0.05, ^##^p < 0.01, ^###^p < 0.001, ^####^p < 0.0001 vs CON; *p < 0.05, **p < 0.01, ***p < 0.001, ****p < 0.0001 vs HFD (the CON, n = 10), HFD (n = 12) and HFD + EGCG (n = 12)

Firstly, compared with normal group, the HFD + EGCG mice had a less body weight (Fig. 1B). We estimated the effects of EGCG on the lipid and glucose metabolism after EGCG administration. Livers were analyzed histologically using H&E and oil red staining. Compared with the control, HFD mice has shown a mount number of lipid droplets in hepatocytes. But these lipid droplets were decreased in both size and number in EGCG + HFD group (Fig. 1K, L). In according with the morphological data, TG and TC level in liver tissue (Fig. 1F, G) and in plasma (Fig. 1D, E) were also obviously decreased by EGCG administration. Serum levels of ALT and AST are typical indicators of biochemical parameters of liver injury, correlated well with the degree of steatosis. The results of serum ALT and AST are shown long time HFD administration elevated the levels of ALT and AST, which could be reversed by EGCG (Fig. 1H, I). By contrast, the glucose response during intraperitoneal glucose tolerance test (IPGTT) significantly improved in the HFD + EGCG group (Fig. 1C). After 16 weeks of treatment, the IPGTT-AUC (area under the curve) showed the HFD + EGCG group was significantly lower than HFD group (Fig. 1J).

### EGCG downregulates lipogenesis-related genes in HFD-fed mice

**Fig. 2:**
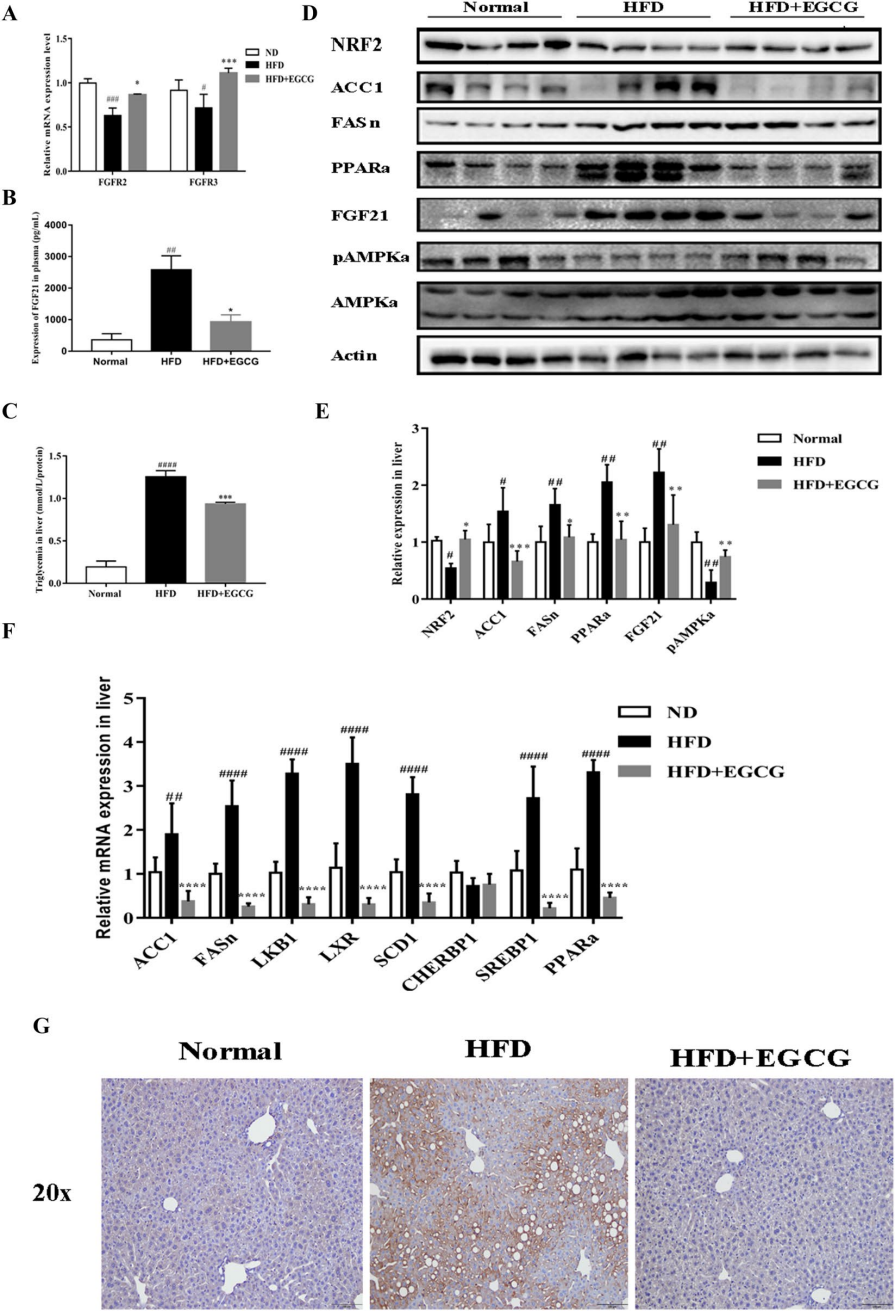
Effects of EGCG on the expression of lipogenesis-related genes and FGF21/AMPK protein in liver tissue. **A** The levels of FGFR 2 and FGFR3. **B** Serum FGF21 levels. **C** TG levels in liver tissue. **D **Expression of PPAR–FGF21–AMPK by Western blotting. **E** Protein densities were measured by Western blotting, **F **Expression levels of lipogenesis-related genes mRNA were determined in liver tissue. **G **Immunohistochemical assay FGF21 of liver; ^#^p < 0.05, ^##^p < 0.01, ^###^p < 0.001, ^####^p < 0.0001 vs CON; *p < 0.05, **p < 0.01, ***p < 0.001, ****p < 0.0001 vs HFD. (the CON, n = 10), HFD (n = 12) and HFD + EGCG (n = 12)

**Fig. 3:**
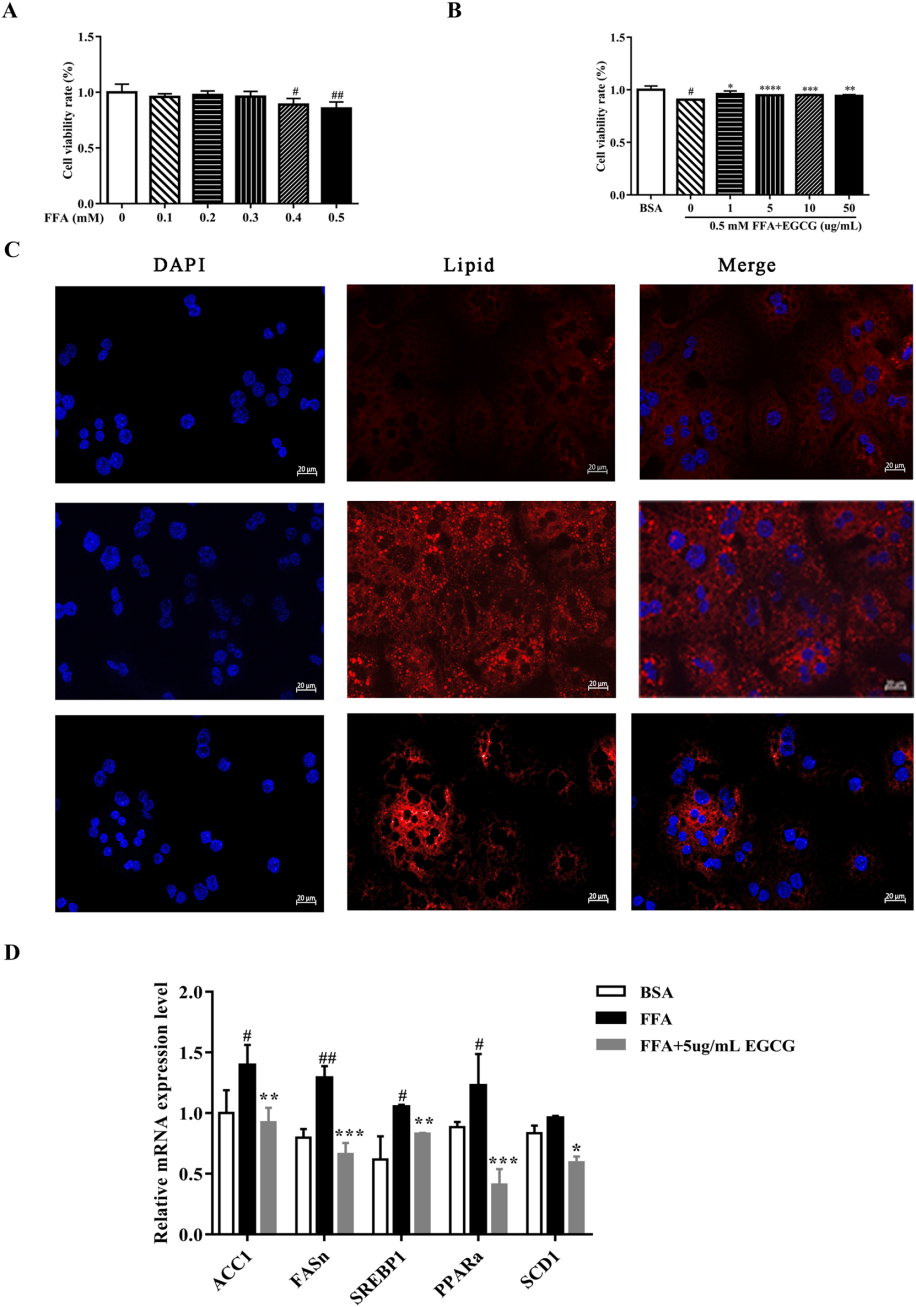
EGCG suppressed FFAs-induced injury in hepatocytes. **A**, **B** CCK8 assay; **C** Immunofluorescence for lipid(× 20); **D** Relative expression of lipogenesis related genes in hepatocytes; Data were presented as mean ± SEM. ^#^p < 0.05, ^##^p < 0.01, ^###^p < 0.001, ^####^p < 0.0001 vs BSA; *p < 0.05, **p < 0.01, ***p < 0.001, ****p < 0.0001 vs FFA. (n = 4)

To illuminate the mechanism of EGCG exerted its antisteatotic, hypolipidemic effects on HFD, we analyzed genes levels of de novo lipogenesis-related genes in the liver. The levels of ACC, fatty acid synthase (FAS), LKB1, LXR, PPARα, stearoyl-CoA desaturase-1 (SCD-1), CHERBP1 and SREBP-1 were higher in the HFD group than the normal, but EGCG administration significantly decreased the levels of these genes except for CHERBP1 gene (Fig. 2F). In consistence with the vivo, the genes of ACC1, FASn, SREBP1, PPARα and SCD1 mRNA were all increased with FFA in primary mouse hepatocyte, but the levels were reversed in EGCG administration in vitro (Fig. 3D).

### EGCG inhibited oxidative stress and increased the level of antioxidant enzymes (SOD) in HFD-fed mice

**Fig. 4:**
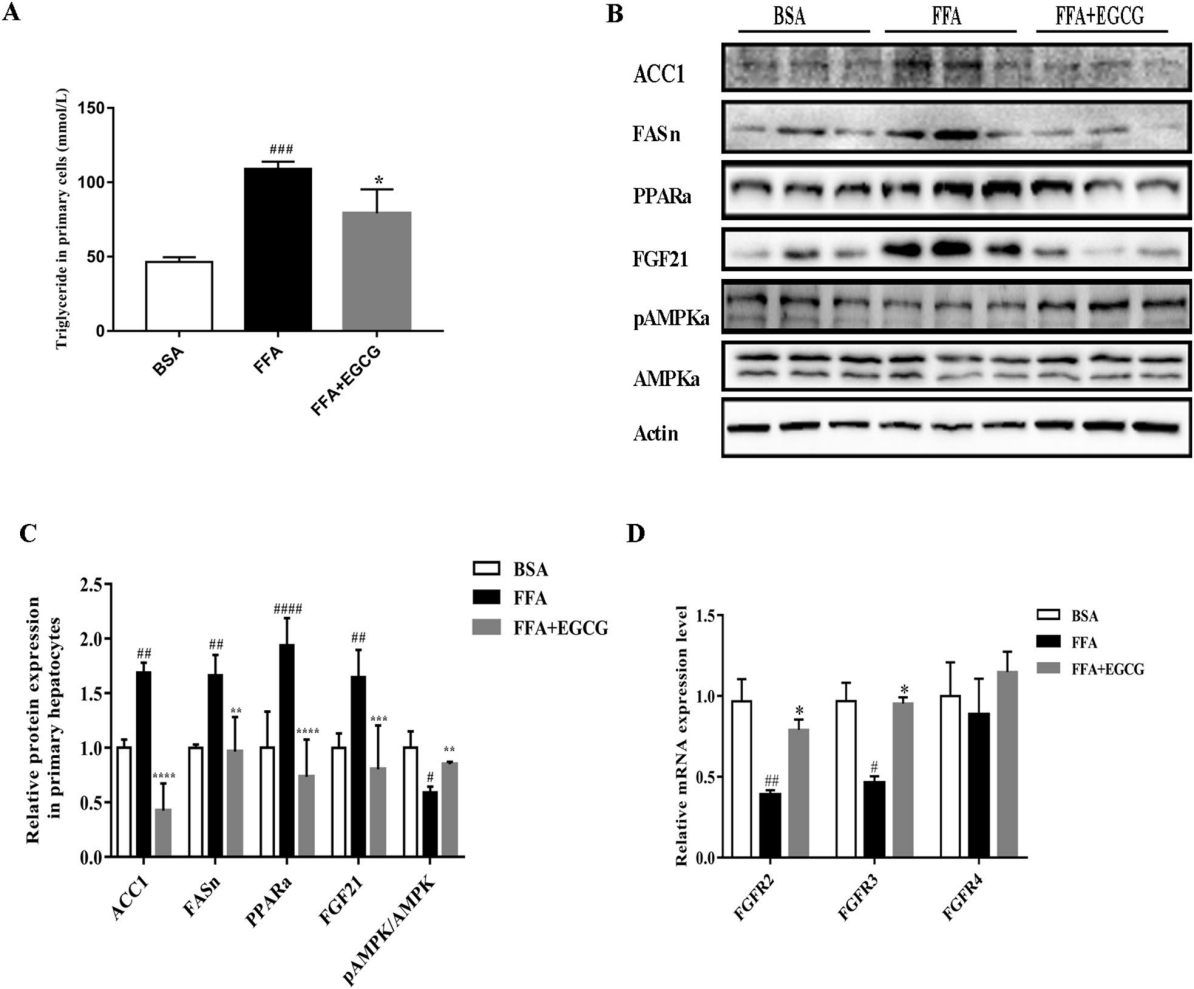
Effects of EGCG on the gene expression and protein in FFAs-induced hepatocytes. **A** TG levels in hepatocytes; **B** effects of EGCG on protein expression of ACC1, FASn, PPAR–FGF21–AMPK; **C** quantification of Fig. **B**; **D **levels of FGFR2,FGFR3 and FGFR4 in hepatocytes; ^#^p < 0.05, ^##^p < 0.01, ^###^p < 0.001, ^####^p < 0.0001 vs BSA; *p < 0.05, **p < 0.01, ***p < 0.001, ****p < 0.0001 vs FFA

To better understand the effect of EGCG on oxidative stress in HFD-Fed Mice, an oxidative stress marker malondialdehyde (MDA), the level of Nrf2 and the level of antioxidant enzymes (SOD) in HFD-induced mice with or without EGCG treatment were measured. We found that the levels of MDA were increased, whereas the antioxidant enzymes SOD and Nrf2 levels were significantly decreased in HFD-induced mice (Figs. 2D, 4A, B). Treatment with EGCG significantly improved HFD-induced hepatic oxidative stress.

### EGCG inhibited lipid accumulation and FFA-induced injury in primary mouse hepatocytes

**Fig. 5:**
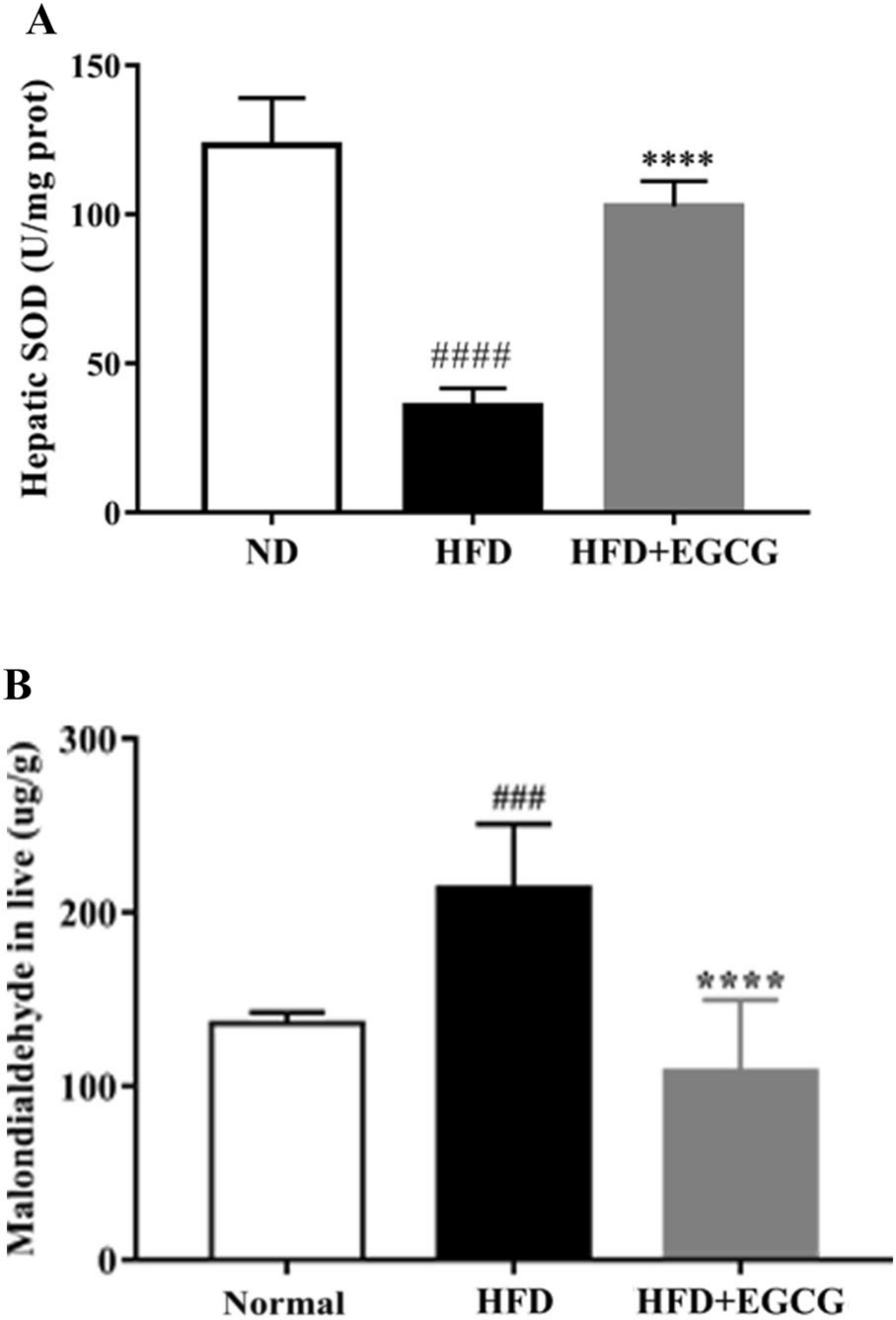
Effects of EGCG on the SOD (**A**) and MDA (**B**) levels in livers. ^#^p < 0.05, ^##^p < 0.01, ^###^p < 0.001, ^####^p < 0.0001 vs CON; *p < 0.05, **p < 0.01, ***p < 0.001, ****p < 0.0001 vs HFD. the CON ( n = 10), HFD (n = 12) and HFD + EGCG (n = 12)

To further study the beneficial mechanism of EGCG in NAFLD, we treated the primary mouse hepatocytes with FFA. We treated the mouse hepatocytes with different doses of FFAs and EGCG for 48 h to measure the effect of FFAs and EGCG using an CCK8 assay (Fig. 3A, B). The cell viability rate is dose-dependent with EGCG administration, the beneficial effects of EGCG was in a dose-dependent manner on hepatocytes. After analyzing the variation tendency, we found EGCG showed the best effect at 0.5 mM. Thus, EGCG was used at the doses of 0.5 mM to treat the cell hepatocytes. The oil red staining results showed that EGCG stimulation could obviously reduce the lipid concentration (Fig. 3C) and TG levels (Fig. 5A) in FFA-treated hepatocytes. These results shown EGCG could protect primary hepatocytes against FFA-induced injury.

### Hfd-fed mice and FFA induced lipid accumulation in cells is inhibited by EGCG through regulation the FGF21–AMPK axis

Based on the beneficial effects of FGF21 mentioned above, we speculated the protective effects of EGCG were closely related to FGF21–FGFR–AMPK axis, so we detected the changes both in vivo and in vitro. Plasma circulating FGF21 levels in HFD-fed mice were increased. However, EGCG can alleviate this increase (Fig. 2B). Meanwhile in liver tissue the FGF21 levels also decreased in immunohistochemistry in EGCG administration (Fig. 2G). EGCG treatment can down-regulate FGF21 expression and up-regulate the FGFR2 and FGFR3 levels not only in liver tissue (Fig. 2A) but also in hepatocytes (Fig. 5D), accordingly activating P-AMPK/AMPK to protect liver against HFD-induced (Fig. 2D) and FFA-induced injury (Fig. 5B). Together, these results suggested that the activation of the FGF21–AMPK pathway is involved in EGCG-induced suppression of lipogenesis.

## Discussion

**Fig. 6:**
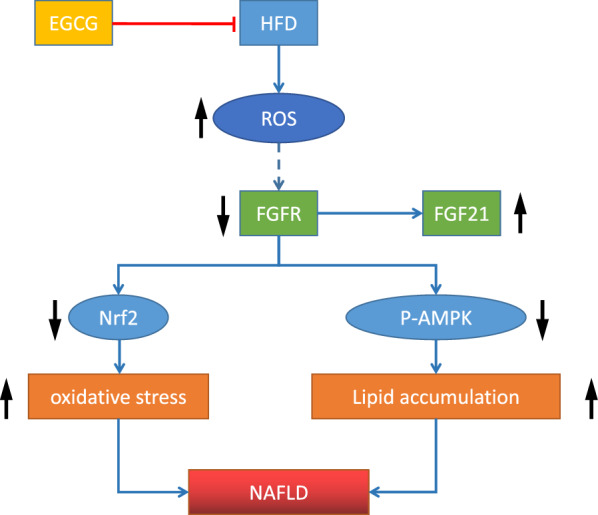
Schematic of the mechanism of EGCG in attenuating NAFLD. Lipotoxicity induced from HFD can induce ROS, and activating ROS and chronic inflammation suppress FGFR expression, leading to a compensatory increase in FGF21 synthesis and secretion. It can inhibit AMPK pathway and Nrf2 pathway. The damage was reversed after administration of EGCG

EGCG plays important biological and pharmacological roles in mammals. Nevertheless, the beneficial effect and mechanism of EGCG in the process of NAFLD are not yet explained. We found that systemic administration of EGCG in HFD group can ameliorate the glucose tolerance, TG and TC levels; accompanied with down-regulating the oxidative stress and the levels of FGF21which alleviated the FGF21 resistance, at the same time increased the levels of FGFR, simultaneously activating the p-AMPKα/AMPKα and downstream genes associated with lipogenesis. These data together indicated that EGCG may become a promising drug to treat or relieve NAFLD (Fig. 6).

Firstly, EGCG administration not only decreased body weight and the levels of TC, TG in plasma and in liver, but also reduced serum glucose level in mice. At the same time, serum levels of ALT and AST are typical indicators of biochemical parameters of liver injury, correlated well with the degree of steatosis. The levels of ALT and AST were decreased in EGCG administration. The results of oil red staining showed EGCG administration alleviated the formation of lipid droplets. In consistent with previous literature, EGCG treatment can inhibit HFD-induced weight compare with HFD group [8]. Our study indicated administration of EGCG significantly alleviated metabolic profile and prevented hepatic fat accumulation in HFD-fed mice.

FGF21 is a hormone-like FGF commonly expressed in the liver, adipose tissue and pancreas [19]. Circulating FGF21 is predominantly liver-derived [19]. As we all known, FGF21 plays important role in many metabolic processes including insulin sensitivity, glucose and lipid metabolism by binding to mainly fibroblast growth factor receptors (FGFRs) and co-receptor β-Klotho, a transmembrane glycoprotein. There are four FGFRs: FGFR1-4 [20, 21]. Recent studies demonstrated FGF21 is regulated by peroxisome proliferator-activated receptor α (PPARα) in WAT and liver [12, 13]. But the observational studies in humans have shown circulating FGF21 levels are elevated in subjects with NAFLD compare with the control [11, 22]. Consistent with the notion the ‘FGF21 resistance’ proposed by Yang et al. to highlight this indicated a state of NAFLD [23]. In the setting of NAFLD, PPARα is activated by higher intrahepatic fatty acids [24] and sustained activation of this dysfunctional PPARα signaling pathway results in the elevated FGF21 levels that are characteristic of individuals with NAFLD [25].

Mechanistically, it appears that the elevation of FGF21 levels in NAFLD patients is the product of dysfunctional PPARα signaling. Mice with NAFLD have an augmented response to PPARα agonism, resulting in enhanced FGF21 expression when compared with non-NAFLD controls. In according with other study, our study also has shown the same results that high PPARα, FGF21 level, and lower FGF receptors level in HFD-fed mice and in cells, indicating the dysfunction of beneficial metabolic effects about FGF21. This phenomenon was reversed after administration of EGCG not only in vivo but also in cells. Meanwhile EGCG can increase the expression of FGF receptors to improve the anti-NAFLD function of FGF21.

It has been shown that AMP-dependent protein kinase α (AMPKα) has been considered as the fuel gauge of the cells or the natural energy sensor that regulates lipid metabolism [14]. Liver kinase B1 (LKB1) accounts for the phosphorylation and activation of AMPK and also is the main upstream kinase. AMPKα plays important roles in regulating de novo lipogenesis in livers and it is acceptable that inhibiting of hepatic lipogenesis for prevention of NAFLD progression has been confirmed as a potential therapeutic target [26]. Usually activated AMPK possess the ability by the phosphorylation and inactivate Acetyl coenzyme A carboxylase (ACC) [27] to increase fatty acid oxidation in the liver, with simultaneous inhibition of hepatic lipogenesis and cholesterol synthesis [28]. Meanwhile, research has shown activation of AMPKα can decrease the sterol element-binding protein 1c (SREBP-1C) carbohydrate response element-binding protein (ChREBP) as a negative regulator [29]. As we all known, SREBP1 is a master transcription factor regulating the expression of genes that control fatty acid and cholesterol synthesis, including acetyl CoA carboxylase (ACC), fatty acid synthase (FAS) and stearoyl-CoA desaturase-1 (SCD1) [30–32]. In the study, AMPK downstream genes (ACC, FASn, SREPB1, ChREBP and SCD1) involved in the de novo lipogenesis pathway also were investigated. These genes in our study has shown EGCG administration can improve lipid metabolism by regulating the lipogenic genes, which in turn alleviate the lipid accumulation of NAFLD. Li et al. EGCG can reduced obesity and white adipose tissue through AMPK activation. Therefore, we examined the activity of LKB-AMPKα pathway in liver tissue. Our results shown that P-AMPKα and LKB were inhibited in HFD-fed group and administration of EGCG can increase p-AMPKα level. This is consistent with another study, which shown that supplement with EGCG stimulates AMPK activation and regulates essential enzymes involved in the de novo lipogenesis pathway in HFD-fed mice.

EGCG is principally claimed as a hepatoprotectant and crucially affects the hepatic system of animals upon oxidative stress. NRF2 is a key factor to limit oxidative stress and it can also alleviate NAFLD through multiple mechanisms, including regulating the expressions of genes regarding pro-inflammatory response and lipid metabolism and mitigating oxidative stress. So we also assessed the antioxidant effect including Nrf2 and antioxidant enzymes SOD in livers. Our study showed HFD can promote the oxidative stress and inhibit Nrf2 protein. And they were reversed after administration of EGCG.

## Conclusion

Taken together, our data suggested that EGCG can alleviate HFD-induced NAFLD through reverse of FGF21 resistance and promotion of FGF21–AMPK pathway and Nrf2-antioxidative roles. EGCG seemed be as an promising options for the treatment of NAFLD.

## Data Availability

Data are available upon reasonable request. Requests to access the datasets should be directed to zhaodong@ccmu.edu.cn.
